# Is the 4C Score Still a Valid Item to Predict In-Hospital Mortality in People with SARS-CoV-2 Infections in the Omicron Variant Era?

**DOI:** 10.3390/life13010183

**Published:** 2023-01-08

**Authors:** Andrea De Vito, Agnese Colpani, Laura Saderi, Mariangela Puci, Beatrice Zauli, Maria Chiara Meloni, Marco Fois, Alessandra Bitti, Cosimo Di Castri, Vito Fiore, Ivana Maida, Sergio Babudieri, Giovanni Sotgiu, Giordano Madeddu

**Affiliations:** 1 Unit of Infectious Diseases, Department of Medicine, Surgery and Pharmacy, University of Sassari, 07100 Sassari, Italy; 2 Clinical Epidemiology and Medical Statistics Unit, Department of Medicine, Surgery and Pharmacy, University of Sassari, 07100 Sassari, Italy

**Keywords:** SARS-CoV-2, COVID-19, 4C score, mortality, predictive score, Omicron variant, vaccine, antiviral treatment

## Abstract

Since the start of the SARS-CoV-2 pandemic, several scores have been proposed to identify infected individuals at a higher risk of progression and death. The most famous is the 4C score. However, it was developed in early 2020. Our study aimed to evaluate the accuracy of the 4C score during the wave in which the Omicron variant was prevalent. An observational study was conducted at an Italian University Hospital between 1 January and 31 July 2022. A receiver operating characteristic (ROC) curve analysis was performed to evaluate the ability of the 4C score to predict mortality. Overall, 1186 people were recruited, of which 160 (13.5%) died. According to the 4C score, 177 (11.6%) were classified as having a low risk of mortality, 302 (25.5%) were intermediate, 596 (50.3%) were high, and 151 (12.7%) were very high. The ROC curve of the 4C score showed an AUC (95% CI) value of 0.78 (0.74–0.82). At the criterion value of > 10, the sensitivity was 76.2% and the specificity was 62.67%. Similar to previous studies, the 4C mortality score performed well in our sample, and it is still a useful tool for clinicians to identify patients with a high risk of progression. However, clinicians must be aware that the mortality rate reported in the original studies was higher than that observed in our study.

## 1. Introduction

Since the beginning of the SARS-CoV-2 pandemic, infected people have reached half a billion with more than six million deaths. Asymptomatic or paucisymptomatic forms of infection occurred in most of the infected individuals [1]. The most prevalent symptoms of coronavirus disease 19 (COVID-19) are a fever, a cough, and dyspnea; a low proportion of patients complain of gastrointestinal symptoms, anosmia, dysgeusia, headaches, and skin lesions [2,3,4]. In addition, the infection can be associated with life-threatening systemic inflammation, respiratory failure, and multiorgan dysfunctions [5]. 

Many treatments have been proposed to reduce the disease progression in people with COVID-19. However, most of them have been rejected after different studies confirmed their futility (e.g., hydroxychloroquine and lopinavir/ritonavir). A massive vaccination campaign drained overcrowded hospitals. More than 12.99 billion doses have been administered since the start of the pandemic. However, vaccine uptake is now dramatically decreasing due to a reduced risk perception and the softening of enforcement policies. As a result, a risk of new waves of hospital congestion is around the corner. Unnecessary hospital admissions carry an increased risk of bed rest, immobilization, and nosocomial infections. 

For this reason, a punctual and reliable classification of patients is mandatory to reduce hospitalization and health-system overloads. Several scores have been proposed to identify infected individuals at a higher risk of progression and death [6,7,8,9]. In September 2020, Knight et al. published their score (the 4C score); this was developed with a cohort of patients recruited from the ISARIC Coronavirus Clinical Characterisation Consortium (ICARIC-4C), which selected patients from 260 hospitals across England, Scotland, and Wales [10]. Jones et al. externally validated the score by using the records of the McMaster Multi-Regional Hospital Coronavirus Registry (COREG), a multicenter data registry from Ontario, Canada [11]. The 4C score is characterized by nine items (i.e., age, sex at birth, number of comorbidities, respiratory rate, peripheral oxygen saturation, Glasgow Coma Scale, urea, and C-reactive protein (CRP)). The score, ranging from 0–21, predicts in-hospital mortality from a low risk to a very high risk. It is easy and quick to apply, and few data are needed. However, the 4C score has many limitations. First, it is limited to determining in-patient hospital mortality and is not designed to be applicable to an outpatient setting. Second, it was developed using data collected between February and May 2020 from infections caused by a wild-type strain that is associated with a more severe disease compared with that caused by the Omicron variant. Third, no vaccines and early therapies (monlupiravir, nirmatrelvir/ritonavir, remdesivir, or monoclonal antibodies) were previously available. Finally, the 4C score considers the burden of comorbidities according to the presence of none (0 points), one (1 point), or more than one (2 points) without differentiating the type of comorbidities. For all these reasons, we believed that an external validation considering the latest changes in epidemiology and available treatments was necessary. Our study aimed to evaluate the accuracy of the 4C score in patients with SARS-CoV-2 infections during the wave in which the Omicron variant was prevalent. 

## 2. Materials and Methods

### 2.1. Study Design and Sample

An observational study was conducted at an Italian University Hospital between 1 January and 31 July 2022. All people included in the study were evaluated by an infectious disease consultant when admitted to the emergency room, an infectious disease ward, or other wards for in-hospital infections. 

The inclusion criteria were: (i) age ≥ 18 years; (ii) confirmed diagnosis of SARS-CoV-2 infection by a polymerase chain reaction (PCR) or third-generation antigenic test; (iii) collection of the variables needed for the computation of the 4C score; and (iv) having at least four weeks of follow-up.

Information on the medical history, symptoms, computer tomography (CT) findings, blood test results, cause/s of hospital admission, disease progression (need for oxygen supplementation, non-invasive or invasive ventilation, or death), and treatment were collected.

### 2.2. Outcome

The primary outcome was all-cause in-hospital mortality. Following the findings of the original manuscripts [10,11], we did not select a limit on the time until death. Therefore, people with more than four weeks of follow-up, but who were hospitalized at the time of the analysis were considered to be alive.

### 2.3. Statistical Analysis

The quantitative variables were summarized with medians and 25–75 percentiles (IQR); the qualitative ones were summarized by absolute and relative (percentage) frequencies. The Shapiro–Wilk test was used to assess the normality of the quantitative data. Differences in the quantitative variables were evaluated using the Mann–Whitney test; Pearson chi-squared or Fisher exact tests were used to assess the differences in the qualitative covariates. A receiver operating characteristic (ROC) curve analysis was performed to evaluate the ability of the 4C score to predict mortality. A two-tailed *p*-value less than 0.05 was considered to be statistically significant. Data analyses were carried out using STATA 17 (StataCorp, TX, USA).

## 3. Results

Overall, 1186 people were recruited. The median (IQR) age was 74 (62–83) years old and 54.3% were males. Of them, 160 (13.5%) died; these were older than those who survived (median (IQR) age of 81.5 (70–88) years old vs. 73 (60–83) years old; *p* < 0.0001) (Table 1).

The patients who died had a higher prevalence of chronic kidney disease, COPD, dementia, hematologic tumors, and cardiovascular diseases. Furthermore, people who died had a lower percentage of a complete vaccination course. The median levels of CRP and urea were significantly higher in the patients who did not survive. In addition, there was a higher percentage of people who complained about dyspnea at the moment of the first evaluation who died. Having ground-glass opacity (GGO) or consolidation at the CT scan were also more common in the group of those who died.

**Figure 1:**
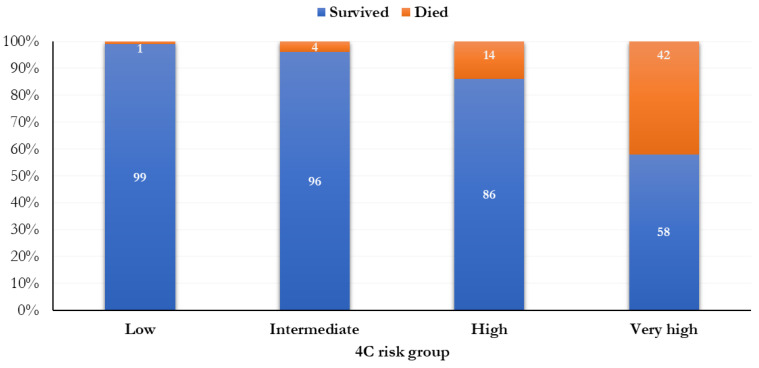
Frequency distribution of the mortality by 4C risk groups.

A total of 137 patients (11.6%) were classified as having a low risk of mortality (0–3 points), 302 (25.5%) were intermediate (4–8 points), 596 (50.3%) were high (9–14 points), and 151 (12.7%) were very high (≥ 15 points) (Figure 1). The mortality was 0.7% (1 person) in people with a low risk of mortality, 4.3% (13 subjects) in those with an intermediate risk of death, 13.9% (83 people) in people with a high risk of death, and 41.7% (63 subjects) in people with a very high risk of death, according to the 4C score.

**Figure 2:**
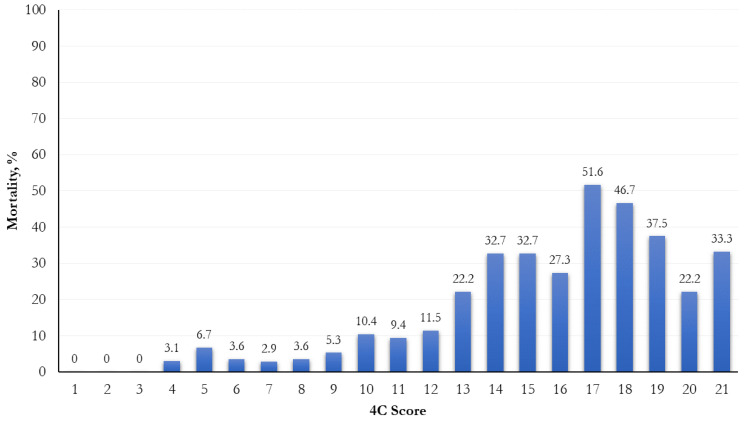
Frequency distribution of mortality by 4C score cut-points.

The mortality was significantly higher when a higher risk score was attributed (*p* < 0.0001; Figure 1). In relation to different 4C score cut-points (range: 0–21), the most increased mortality (51.6%) was observed in the 17th cut-point (Figure 2). 

**Figure 3:**
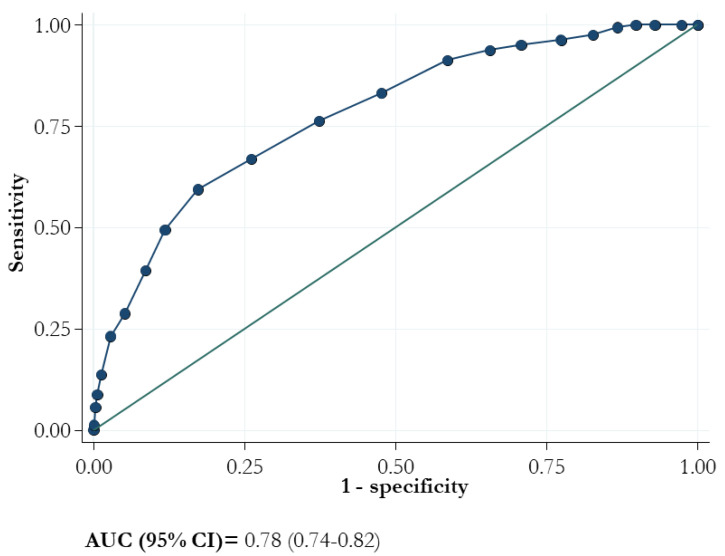
Area under the receiver operating characteristic curve (AUC) for mortality and 4C score.

The ROC curve of the 4C score showed an AUC (95% CI) value of 0.78 (0.74–0.82; Figure 3).

The sensitivity of the model for each value of 4C score ranged from 100.0 to 28.75% whereas the specificity ranged from 0 to 94.83%, respectively (Table 2). At the criterion value of > 10, the sensitivity was 76.2% and the specificity was 62.67%. The lower two cut-offs (3 and 8) demonstrated negative likelihood ratios of 0.047 and 0.21, respectively, and the positive likelihood ratios exceeded 4 for values higher than 13.

Finally, we divided the subjects, depending on whether they had received an early antiviral treatment or not (Table 3). Those who received an early antiviral treatment had a lower mortality than those who did not.

## 4. Discussion

The aim of our study was to evaluate the ability of the 4C score to predict mortality in COVID-19 patients. Our findings suggested that the 4C mortality score is a useful tool for COVID-19 patients admitted to hospital wards.

As described by Jones et al., we confirmed that mortality was higher with increasing 4C scores. Moreover, although the AUC was not high (0.78), its value was acceptable and similar to previous studies [10,11].

**Figure 4:**
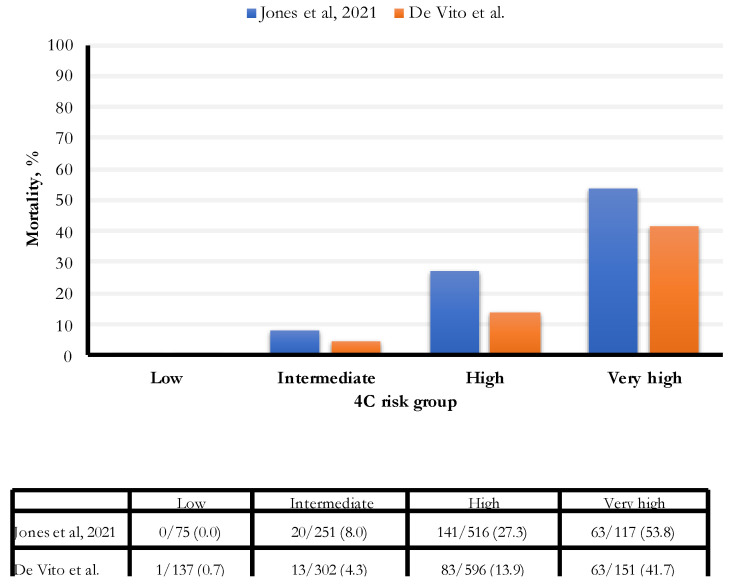
Comparison of 4C score categories between Jones et al. [11] and De Vito et al. cohorts.

In our study, the hospital all-cause mortality was 13.5%, an estimate lower than those found in two recent studies (16.9% and 23.4%, respectively, Gordon and Jones) (Figure 4). In particular, the percentage of people with an intermediate risk was 46% lower (8% vs. 4.3%) in our study; for people with a high risk of death, the percentage was 49% lower (27.3% vs. 13.9%). Finally, for people with a very high risk of death, the rate was 22.5% lower (53.8% vs. 41.7%). The established cut-off for a very high risk of death was 15 points; we observed the most increased death risk for 17 points and above.

These differences could be explained by the data collection period. During these two years, substantial changes in the management of COVID-19 occurred. In addition, several vaccines were commercially distributed, reducing the risk of severe illness and death [12]. In this regard, Watson et al. created a mathematical model to analyze the impact of the anti-SARS-CoV-2 vaccination program considering data from 185 countries. As a result, they estimated that vaccinations prevented 14.4 million deaths globally, confirming that vaccination altered the course of the pandemic [13]. 

Moreover, the principal strain present in Italy was B.1.1.529 (Omicron) and its subvariants during the study period; Omicron has been proven to cause a lower incidence of severe disease and mortality compared with previous variants [14,15].

In addition, three different antiviral therapies have now been approved to avoid the progression of the disease: monlupiravir, nirmatrelvir/ritonavir, and remdesivir [16,17,18,19]. Monlupiravir, a small-molecule ribonucleoside prodrug of hydroxy-cytidine (NHC) with a high genetic barrier [20], was demonstrated to have high efficacy in reducing the disease progression in a clinical trial [16]. Although the chosen population was young in the clinical trial and had a lower comorbidity burden, real-life studies confirmed the efficacy and safety of monlupiravir [21,22,23]. Nirmatrelvir/ritonavir(r) is a protease inhibitor targeting the SARS-CoV-2 3-chymotrypsin-like cysteine protease enzyme (M pro). This target enzyme is essential for viral replication [24]. However, nirmatrelvir is metabolized by CYP3A4; thus, to increase the T_1/2_, it is associated with a booster (ritonavir) that enhances the nirmatrelvir/r. On the other hand, the addition of ritonavir causes various drug–drug interactions. The clinical trial showed excellent efficacy and safety, confirmed by real-life studies [17,22]. Remdesivir is a direct-acting nucleotide prodrug inhibitor of the SARS-CoV-2 RNA-dependent RNA polymerase. It was the first antiviral approved for treating hospitalized patients who needed oxygen supplementation and who had confirmed evidence of COVID-19-related pneumonia. In January 2022, the results of the PINETREE trial were published, demonstrating how a short dose of remdesivir (3 days instead of 5) could reduce the disease progression and hospitalization by 87% with good safety [18]. For these reasons, we believe that the use of these treatments has had a significant impact on reducing the number of people who experienced the disease progression and who died from COVID-19. Furthermore, during the period of our study, monoclonal antibodies were available. The available treatments were casirivimab/imdevimab (Ronapreve**^®^**), tixagevimab/cilgavimab (Evusheld**^®^**), and sotrovimab (Xevudy^®^) [25,26,27]. However, the efficacy of casirivimab/imdevimab and sotrovimab in preventing the disease progression of people infected by the Omicron variant and its subvariants (BA.2, BA.4, and BA.5) is unclear [28]; however, the data about the use of tixagevimab/cilgavimab are more reassuring. In our center, tixagevimab/cilgavimab was not available during the months of our study. Several drugs for patients with severe COVID-19 can also reduce mortality [29,30,31,32,33]. As shown in Table 2, people treated with an early antiviral treatment had a lower mortality rate than those who were not. For this reason, new scores that consider these treatments are needed.

An important limitation of our study was related to its monocentric nature. However, we believe it could be generalized to all countries where antiviral and monoclonal antibodies are available. Another significant limitation was that the scores were calculated retrospectively and not at the evaluation time. The situation is ever-changing, and we will continue to update our tools according to the evolving ecology and management of the infection.

## 5. Conclusions

Similar to previous studies, the 4C mortality score performed well in our sample, and it is still a useful tool for clinicians to identify patients with a high risk of progression. However, clinicians must be aware that the mortality rate reported in the original studies was higher than that observed in our study. Finally, new scores for predicting disease progression that consider early antiviral treatments and vaccinations are needed.

## Figures and Tables

**Table 1:** Characteristics of 1186 people with SARS-CoV-2 infections stratified by survival.

Variables			Total (*n* = 1186)	Survived (*n* = 1026)	Died (*n* = 160)	*p*-Value
Males, n (%)			644 (54.3)	545 (53.1)	99 (61.9)	0.04
Median (IQR) age, years			74 (62–83)	73 (60–83)	81.5 (70–88)	<0.0001
Median (IQR) 4C score			10 (6–12)	9 (6–12)	13 (11–16)	<0.0001
4C risk groups		Low (0–3)	137 (11.6)	136 (13.3)	1 (0.6)	<0.0001
		Intermediate (4–8)	302 (25.5)	289 (28.2)	13 (8.1)	
		High (9–14)	596 (50.3)	513 (50.0)	83 (51.9)	
		Very high (≥ 15)	151 (12.7)	88 (8.6)	63 (39.4)	
Respiratory rate		< 20	788 (66.4)	723 (70.5)	65 (40.6)	<0.0001
		20–29	296 (25.0)	241 (23.5)	55 (34.4)	
		≥ 30	102 (8.6)	62 (6.0)	102 (8.6)	
Glasgow Coma Scale < 15			219 (18.5)	156 (15.2)	63 (39.4)	<0.0001
Peripheral oxygen < 92%			218 (18.4)	155 (15.1)	63 (39.4)	<0.0001
BMI > 30 kg/m^2^, n (%)			291 (24.5)	251 (24.5)	40 (25.0)	0.88
Chronic renal disease, n (%)			195 (16.4)	155 (15.1)	40 (25.0)	0.002
Rheumatological disease, n (%)			63 (5.3)	60 (5.9)	3 (1.9)	0.04
Decompensated diabetes, n (%)			180 (15.2)	149 (14.5)	31 (19.4)	0.11
Diabetes, n (%)			264 (22.3)	224 (21.8)	40 (25.0)	0.37
Chronic liver disease, n (%)			67 (5.7)	54 (5.3)	13 (8.1)	0.14
COPD/emphysema, n (%)			237 (20.0)	193 (18.8)	44 (27.5)	0.01
Hemoglobinopathies, n (%)			5 (0.4)	5 (0.5)	0 (0.0)	1.00
Neurodevelopmental/neurodegenerative diseases, n (%)			315 (26.6)	251 (24.5)	64 (40.0)	<0.0001
Dementia, n (%)			177 (14.9)	132 (12.9)	45 (28.1)	<0.0001
Cerebrovascular events, n (%)			133 (11.2)	110 (10.7)	23 (14.4)	0.17
Oncological disease, n (%)			209 (17.6)	177 (17.3)	32 (20.0)	0.40
Hematological tumors, n (%)			76 (6.4)	59 (5.8)	17 (10.6)	0.02
Solid tumors in chemotherapy, n (%)			33 (2.8)	29 (2.8)	4 (2.5)	1.00
Hematological tumors in chemotherapy, n (%)			55 (4.6)	42 (4.1)	13 (8.1)	0.02
Cardiovascular diseases, n (%)			452 (38.1)	366 (35.7)	86 (53.8)	<0.0001
Heart failure, n (%)			403 (34.0)	321 (31.4)	82 (51.3)	<0.0001
Previous myocardial infarction, n (%)			146 (12.3)	126 (12.3)	20 (12.5)	0.94
Median (IQR) number of comorbidities			2 (1–3)	2 (1–3)	3 (2–4)	<0.0001
Vaccine, n (%)			938 (79.1)	826 (80.5)	112 (70.0)	0.002
N. Vaccine doses, n (%)	0		248 (20.9)	200 (19.5)	48 (30.0)	0.02
	1		32 (2.7)	26 (2.5)	6 (3.8)	
	2		208 (17.5)	179 (17.5)	29 (18.1)	
	3		676 (57.0)	601 (58.6)	75 (46.9)	
	4		22 (1.9)	20 (2.0)	2 (1.3)	
Median (IQR) time, last dose			128.5 (76–186)	127 (76–181)	151.5 (81–223.5)	0.02
In-hospital infection, n (%)			256 (21.6)	211 (20.6)	45 (28.1)	0.03
**Symptoms**						
Fever, n (%)			569 (48.0)	486 (47.4)	83 (51.9)	0.29
Cough, n (%)			455 (38.4)	383 (37.3)	72 (45.0)	0.06
Pharyngodynia, n (%)			160 (13.5)	146 (14.2)	14 (8.8)	0.06
Asthenia, n (%)			389 (32.8)	330 (32.2)	59 (36.9)	0.24
Headache, n (%)			136 (11.5)	125 (12.2)	11 (6.9)	0.05
Myalgia, n (%)			184 (15.5)	160 (15.6)	24 (15.0)	0.85
Gastrointestinal symptoms, n (%)			167 (14.1)	149 (14.5)	18 (11.3)	0.27
Dyspnea, n (%)			380 (32.0)	292 (28.5)	88 (55.0)	<0.0001
Anosmia, n (%)			22 (1.9)	19 (1.9)	3 (1.9)	1.00
**CT findings**						
GGO, n (%)			451 (39.8)	355 (36.4)	96 (60.8)	<0.0001
Consolidation, n (%)			275 (24.3)	206 (21.1)	69 (43.7)	<0.0001
Pulmonary embolism, n (%)			25 (2.1)	21 (2.1)	4 (2.5)	0.77
**Laboratory examination**						
Median (IQR) urea			37 (28–60)	35 (27–55)	60.5 (36.5–110.0)	<0.0001
Median (IQR) CRP			3 (1.3–7.7)	2.5 (1.1–6.6)	8.6 (3.8–16.0)	<0.0001

IQR: interquartile range; BMI: body mass index; COPD: chronic obstructive pulmonary disease; CT: computer tomography; GGO: ground-glass opacity; CRP: C-reactive protein.

**Table 2:** Criterion values and coordinates of the ROC curve.

Criterion	SE	95% CI	SP	95% CI	+LR	95% CI	–LR	95% CI
≥ 0	100.00	97.7–100.0	0.00	0.0–0.4	1.00	1.00–1.00	-	-
> 0	100.00	97.7–100.0	2.63	1.7–3.8	1.03	1.02–1.04	0.00	-
> 1	100.00	97.7–100.0	7.12	5.6–8.9	1.08	1.06–1.09	0.00	-
> 2	100.00	97.7–100.0	10.23	8.4–12.3	1.11	1.09–1.14	0.00	-
> 3	99.37	96.6–100.0	13.26	11.2–15.5	1.15	1.12–1.18	0.047	0.01–0.33
> 4	97.50	93.7–99.3	17.35	15.1–19.8	1.18	1.14–1.22	0.14	0.05–0.38
> 5	96.25	92.0–98.6	22.61	20.1–25.3	1.24	1.19–1.30	0.17	0.08–0.37
> 6	95.00	90.4–97.8	29.24	26.5–32.1	1.34	1.27–1.42	0.17	0.09–0.34
> 7	93.75	88.8–97.0	34.41	31.5–37.4	1.43	1.35–1.52	0.18	0.10–0.33
> 8	91.25	85.8–95.1	41.42	38.4–44.5	1.56	1.45–1.67	0.21	0.13–0.35
> 9	83.12	76.4–88.6	52.34	49.2–55.4	1.74	1.59–1.92	0.32	0.23–0.46
> 10	76.25	68.9–82.6	62.67	59.6–65.6	2.04	1.82–2.30	0.38	0.29–0.50
> 11	66.87	59.0–74.1	73.88	71.1–76.5	2.56	2.20–2.97	0.45	0.36–0.56
> 12	59.38	51.3–67.1	82.75	80.3–85.0	3.44	2.86–4.14	0.49	0.41–0.59
> 13	49.38	41.4–57.4	88.21	86.1–90.1	4.19	3.33–5.27	0.57	0.49–0.67
> 14	39.38	31.8–47.4	91.42	89.5–93.1	4.59	3.48–6.06	0.66	0.58–0.75
> 15	28.75	21.9–36.4	94.83	93.3–96.1	5.57	3.89–7.96	0.75	0.68–0.83

SE: sensitivity; SP: specificity; CI confidence interval.

**Table 3:** Comparison of 4C score and mortality between people who received an early antiviral treatment and people who did not.

	Low	Intermediate	High	Very High
Early Antiviral Treatment	1/29 (3.4)	2/109 (1.8)	16/177 (9.0)	4/13 (30.8)
No Antiviral Treatment	0/108 (0.0)	11/193 (5.7)	67/419 (16.0)	59/138 (42.7)
Overall	1/137 (0.7)	13/302 (4.3)	83/596 (13.9)	63/151 (41.7)

## Data Availability

The data that support the findings of this study are available from the corresponding author, upon reasonable request.

## References

[1] He J., Guo Y., Mao R., Zhang J. (2020). Proportion of asymptomatic coronavirus disease 2019 (COVID-19): A systematic review and meta-analysis. J. Med. Virol..

[2] De Vito A., Fiore V., Princic E., Geremia N., Napodano C.M.P., Muredda A.A., Maida I., Madeddu G., Babudieri S. (2021). Predictors of infection, symptoms development, and mortality in people with SARS-CoV-2 living in retirement nursing homes. PLoS ONE.

[3] Geremia N., De Vito A., Gunnella S., Fiore V., Princic E., Napodano C.P., Madeddu G., Babudieri S. (2020). A Case of Vasculitis-Like Skin Eruption Associated with COVID-19. Infect. Dis. Clin. Pract..

[4] Vaira L.A., De Vito A., Lechien J.R., Chiesa-Estomba C.M., Mayo-Yàñez M., Calvo-Henrìquez C., Saussez S., Madeddu G., Babudieri S., Boscolo-Rizzo P. (2022). New Onset of Smell and Taste Loss Are Common Findings Also in Patients with Symptomatic COVID-19 after Complete Vaccination. Laryngoscope.

[5] Iba T., Connors J.M., Levy J.H. (2020). The coagulopathy, endotheliopathy, and vasculitis of COVID-19. Inflamm. Res..

[6] King J.T., Yoon J.S., Rentsch C.T., Tate J.P., Park L.S., Kidwai-Khan F., Skanderson M., Hauser R.G., Jacobson D.A., Erdos J. (2020). Development and validation of a 30-day mortality index based on pre-existing medical administrative data from 13,323 COVID-19 patients: The Veterans Health Administration COVID-19 (VACO) Index. PLoS ONE.

[7] Garibaldi B.T., Fiksel J., Muschelli J., Robinson M.L., Rouhizadeh M., Perin J., Schumock G., Nagy P., Gray J.H., Malapati H. (2021). Patient Trajectories among Persons Hospitalized for COVID-19: A Cohort Study. Ann. Intern. Med..

[8] Goodacre S., Thomas B., Sutton L., Burnsall M., Lee E., Bradburn M., Loban A., Waterhouse S., Simmonds R., Biggs K. (2021). Derivation and validation of a clinical severity score for acutely ill adults with suspected COVID-19: The PRIEST observational cohort study. PLoS ONE.

[9] Wynants L., Van Calster B., Collins G.S., Riley R.D., Heinze G., Schuit E., Bonten M.M.J., Dahly D.L., Damen J.A., Debray T.P.A. (2020). Prediction models for diagnosis and prognosis of covid-19: Systematic review and critical appraisal. BMJ.

[10] Knight S.R., Ho A., Pius R., Buchan I., Carson G., Drake T.M., Dunning J., Fairfield C.J., Gamble C., Green C.A. (2020). Risk stratification of patients admitted to hospital with covid-19 using the ISARIC WHO Clinical Characterisation Protocol: Development and validation of the 4C Mortality Score. BMJ.

[11] Jones A., Pitre T., Junek M., Kapralik J., Patel R., Feng E., Dawson L., Tsang J.L.Y., Duong M., Ho T. (2021). External validation of the 4C mortality score among COVID-19 patients admitted to hospital in Ontario, Canada: A retrospective study. Sci. Rep..

[12] Chi W.-Y., Li Y.-D., Huang H.-C., Chan T.E.H., Chow S.-Y., Su J.-H., Ferrall L., Hung C.-F., Wu T.-C. (2022). COVID-19 vaccine update: Vaccine effectiveness, SARS-CoV-2 variants, boosters, adverse effects, and immune correlates of protection. J. Biomed. Sci..

[13] Watson O.J., Barnsley G., Toor J., Hogan A.B., Winskill P., Ghani A.C. (2022). Global impact of the first year of COVID-19 vaccination: A mathematical modelling study. Lancet Infect. Dis..

[14] Nyberg T., Ferguson N.M., Nash S.G., Webster H.H., Flaxman S., Andrews N., Hinsley W., Bernal J.L., Kall M., Bhatt S. (2022). Comparative analysis of the risks of hospitalisation and death associated with SARS-CoV-2 omicron (B.1.1.529) and delta (B.1.617.2) variants in England: A cohort study. Lancet.

[15] Ughi N., Bernasconi D.P., Del Gaudio F., Dicuonzo A., Maloberti A., Giannattasio C., Tarsia P., Puoti M., Scaglione F., Beltrami L. (2022). Trends in all-cause mortality of hospitalized patients due to SARS-CoV-2 infection from a monocentric cohort in Milan (Lombardy, Italy). J. Public Health.

[16] Jayk Bernal A., Gomes da Silva M.M., Musungaie D.B., Kovalchuk E., Gonzalez A., Delos Reyes V., Martín-Quirós A., Caraco Y., Williams-Diaz A., Brown M.L. (2022). Molnupiravir for Oral Treatment of Covid-19 in Nonhospitalized Patients. N. Engl. J. Med..

[17] Hammond J., Leister-Tebbe H., Gardner A., Abreu P., Bao W., Wisemandle W., Baniecki M., Hendrick V.M., Damle B., Simón-Campos A. (2022). Oral Nirmatrelvir for High-Risk, Nonhospitalized Adults with Covid-19. N. Engl. J. Med..

[18] Gottlieb R.L., Vaca C.E., Paredes R., Mera J., Webb B.J., Perez G., Oguchi G., Ryan P., Nielsen B.U., Brown M. (2022). Early Remdesivir to Prevent Progression to Severe Covid-19 in Outpatients. N. Engl. J. Med..

[19] De Vito A., Colpani A., Saderi L., Puci M., Zauli B., Fiore V., Fois M., Meloni M.C., Bitti A., Di Castri C. (2023). Impact of Early SARS-CoV-2 Antiviral Therapy on Disease Progression. Viruses.

[20] Malone B., Campbell E.A. (2021). Molnupiravir: Coding for catastrophe. Nat. Struct. Mol. Biol..

[21] De Vito A., Colpani A., Bitti A., Zauli B., Meloni M.C., Fois M., Denti L., Bacciu S., Marcia C., Maida I. (2022). Safety and efficacy of molnupiravir in SARS-CoV-2 infected patients: A real-life experience. J. Med. Virol..

[22] Gentile I., Scotto R., Shiano Moriello N., Pinchera B., Villari R., Trucillo E., Ametrano L., Fusco L., Castaldo G., Buonomo A.R. (2022). Nirmatrelvir/Ritonavir and Molnupiravir in the Treatment of Mild/Moderate COVID-19: Results of a Real-Life Study. Vaccines.

[23] Wong C.K.H., Au I.C.H., Lau K.T.K., Lau E.H.Y., Cowling B.J., Leung G.M. (2022). Real-world effectiveness of molnupiravir and nirmatrelvir plus ritonavir against mortality, hospitalisation, and in-hospital outcomes among community-dwelling, ambulatory patients with confirmed SARS-CoV-2 infection during the omicron wave in Hong Kong: An observational study. Lancet.

[24] Saravolatz L.D., Depcinski S., Sharma M. (2022). Molnupiravir and Nirmatrelvir-Ritonavir: Oral COVID Antiviral Drugs. Clin. Infect. Dis..

[25] Abani O., Abbas A., Abbas F., Abbas M., Abbasi S., Abbass H., Abbott A., Abdallah N., Abdelaziz A., Abdelfattah M. (2022). Casirivimab and imdevimab in patients admitted to hospital with COVID-19 (RECOVERY): A randomised, controlled, open-label, platform trial. Lancet.

[26] Gupta A., Gonzalez-Rojas Y., Juarez E., Casal M.C., Moya J., Falci D.R., Sarkis E., Solis J., Zheng H., Scott N. (2022). Effect of Sotrovimab on Hospitalization or Death Among High-risk Patients with Mild to Moderate COVID-19: A Randomized Clinical Trial. JAMA.

[27] Ginde A.A., Paredes R., Murray T.A., Engen N., Grandits G., Vekstein A., Ivey N., Mourad A., Sandkovsky U., Gottlieb R.L. (2022). Tixagevimab-cilgavimab for treatment of patients hospitalised with COVID-19: A randomised, double-blind, phase 3 trial. Lancet Respir. Med..

[28] Takashita E., Yamayoshi S., Simon V., van Bakel H., Sordillo E.M., Pekosz A., Fukushi S., Suzuki T., Maeda K., Halfmann P. (2022). Efficacy of Antibodies and Antiviral Drugs against Omicron BA.2.12.1, BA.4, and BA.5 Subvariants. N. Engl. J. Med..

[29] De Vito A., Poliseno M., Colpani A., Zauli B., Puci M.V., Santantonio T., Meloni M.C., Fois M., Fanelli C., Saderi L. (2022). Reduced risk of death in people with SARS-CoV-2 infection treated with remdesivir: A nested case-control study. Curr. Med. Res. Opin..

[30] De Vito A., Saderi L., Fiore V., Geremia N., Princic E., Fanelli C., Muredda A.A., Napodano C.P., Moi G., Maida I. (2022). Early treatment with low-molecular-weight heparin reduces mortality rate in SARS-CoV-2 patients. Panminerva Med..

[31] Rodriguez-Guerra M., Jadhav P., Vittorio T.J. (2021). Current treatment in COVID-19 disease: A rapid review. Drugs Context.

[32] Mazzitelli M., Arrighi E., Serapide F., Pelle M.C., Tassone B., Lionello R., Marrazzo G., Laganà D., Costanzo F.S., Matera G. (2021). Use of subcutaneous tocilizumab in patients with COVID-19 pneumonia. J. Med. Virol..

[33] Balena F., Bavaro D.F., Fabrizio C., Bottalico I.F., Calamo A., Santoro C.R., Brindicci G., Bruno G., Mastroianni A., Greco S. (2020). Tocilizumab and corticosteroids for COVID-19 treatment in elderly patients. J. Gerontol. Geriatr..

